# Pioglitazone Improves *In Vitro* Viability and Function of Endothelial Progenitor Cells from Individuals with Impaired Glucose Tolerance

**DOI:** 10.1371/journal.pone.0048283

**Published:** 2012-11-05

**Authors:** Valentina Spigoni, Angela Picconi, Monia Cito, Valentina Ridolfi, Sabrina Bonomini, Chiara Casali, Ivana Zavaroni, Luigi Gnudi, Marco Metra, Alessandra Dei Cas

**Affiliations:** 1 Cardiology, Department of Experimental and Applied Medicine, University of Brescia, Brescia, Italy; 2 Endocrinology, Department of Internal Medicine and Biomedical Sciences, University of Parma, Parma, Italy; 3 Cardiovascular Division, King’s College London, London, United Kingdom; University of Padova, Italy

## Abstract

**Background:**

Evidence suggests that the PPARγ-agonist insulin sensitizer pioglitazone, may provide potential beneficial cardiovascular (CV) effects beyond its anti-hyperglycaemic function. A reduced endothelial progenitor cell (EPC) number is associated with impaired glucose tolerance (IGT) or diabetes, conditions characterised by increased CV risk.

**Aim:**

To evaluate whether pioglitazone can provide benefit *in vitro* in EPCs obtained from IGT subjects.

**Materials and Methods:**

Early and late-outgrowth EPCs were obtained from peripheral blood mononuclear cells of 14 IGT subjects. The *in vitro* effect of pioglitazone (10 µM) with/without PPARγ-antagonist GW9662 (1 µM) was assessed on EPC viability, apoptosis, ability to form tubular-like structures and pro-inflammatory molecule expression.

**Results:**

Pioglitazone increased early and late-outgrowth EPC viability, with negligible effects on apoptosis. The capacity of EPCs to form tubular-like structures was improved by pioglitazone in early (mean increase 28%; p = 0.005) and late-outgrowth (mean increase 30%; p = 0.037) EPCs. Pioglitazone reduced ICAM-1 and VCAM-1 adhesion molecule expression in both early (p = 0.001 and p = 0.012 respectively) and late-outgrowth (p = 0.047 and p = 0.048, respectively) EPCs. Similarly, pioglitazone reduced TNFα gene and protein expression in both early (p = 0.034;p = 0.022) and late-outgrowth (p = 0.026;p = 0.017) EPCs compared to control. These effects were prevented by incubation with the PPARγ-antagonist GW9662.

**Conclusion:**

Pioglitazone exerts beneficial effects *in vitro* on EPCs isolated from IGT subjects, supporting the potential implication of pioglitazone as a CV protective agents.

## Introduction

The peroxisome proliferator-activated receptor-gamma (PPARγ) agonist pioglitazone is an insulin-sensitizing agent that is currently used in the treatment of type 2 diabetes mellitus. Pioglitazone has also been shown to exert favourable cardiovascular (CV) effects in slowing atherosclerosis progression [1] and may reduce the risk of myocardial infarction, stroke and premature death in high risk diabetic patients [2]. Impaired glucose tolerance (IGT) is a prediabetic condition characterized by insulin-resistance, predisposing to increased cardiovascular disease (CVD) risk [3]. Recent studies have demonstrated that pioglitazone reduces atherosclerotic plaque inflammation [4] and development [5], [6], and improves endothelial function [7] in subjects with IGT, pointing towards potential beneficial CV properties in pre-diabetic conditions. Experimental studies support the hypothesis that putative pioglitazone CV protective effects extend beyond its metabolic action [8], [9], although the precise molecular mechanisms remain to be elucidated. Robust evidence has demonstrated that CV function and angiogenesis are significantly modulated by endothelial progenitor cells (EPCs), a subset of bone marrow-derived stem cells [10] that play a critical role in the maintenance of endothelial homeostasis contributing to vessel repair following endothelial damage [11]. Reduced EPC number and function are associated with the presence of traditional CV risk factors and with the development of atherosclerosis [12], [13], suggesting that endothelial injury, in the absence of sufficient circulating EPCs, promotes progression of vascular disease. In humans, the number of circulating EPCs is reduced in diabetes [14], metabolic syndrome [15] and insulin resistance [16], [17]. EPCs can be isolated and differentiated *ex vivo* from the circulating mononuclear cell fraction and show specific endothelial markers and properties. Currently, two cell subpopulations, both capable of mediating angiogenesis, have been described and isolated: early (or circulating angiogenic cells CAC) and late-outgrowth EPCs [18], [19].

Clinical studies have demonstrated that pioglitazone improves the number and migratory capacity of EPCs isolated from diabetic subjects [20] and from patients with coronary artery disease (CAD) and normal glucose tolerance [21], a phenomenon paralleled by a reduction in circulating inflammatory markers. Pioglitazone anti-inflammatory effects have been demonstrated in human studies [20], [21] and *in vitro* in activated endothelial cells [22]. However, the capacity of EPCs to form tubular-like structures and the expression of inflammatory molecules in EPCs in the presence of pioglitazone remains to be investigated. To date, no studies are available that have examined the direct effects of pioglitazone on EPCs isolated from IGT subjects. Therefore, the aim of our study was to evaluate whether the addition of pioglitazone *in vitro* can prove benefit in EPC obtained from IGT subjects in terms of apoptosis, viability and tube formation capacity. We also sought to investigate *in vitro* potential changes in EPC pro-inflammatory molecule expression in the presence of pioglitazone. This preclinical study shows that pioglitazone improves *in vitro* angiogenic capacity of EPCs isolated from IGT subjects and reduces inflammation.

**Table 1:** Clinical characteristics of the study population.

Characteristics	
Age (yrs)	58±6
Gender (M/F)	7/7
Cigarette smoking (yes/no)	2/12
Plasma Glucose (mg/dl)	120±18
HbA_1C_ (%)	5.9±0.6
Insulin (µU/ml)	6.2±2.7
Total cholesterol (mg/dl)	200±26
HDL-cholesterol (mg/dl)	45±14
LDL-cholesterol (mg/dl)	134±22
Triglycerides (mg/dl)	104(77–163)
Creatinine (mg/dl)	0.92±0.16
GOT (U/L)	24±10
GPT (U/L)	28±15
Waist circumference (cm)	94±9
BMI (kg/m^2^)	26.1±3.1
SBP (mmHg)	125±8
DBP (mmHg)	76±4

Data expressed as mean±SD or N. Laboratory and diagnostic assays were from fasted blood samples.

HbA_1c_ = glycated hemoglobin;

HDL = high-denisty lipoprotein;

LDL = low-density lipoprotein;

GOT = glutamic-oxaloacetic transaminase;

GPT = glutamic-pyruvic transaminase;

BMI = body mass index;

SBP = systolic blood pressure;

DBP = diastolic blood pressure.

Cigarette smoking refers to current cigarette smoker.

## Materials and Methods

### Ethics Statement

The study protocol was in accordance with the Declaration of Helsinki and was approved by the Ethical Committee of Parma University. A written informed consent was obtained from all subjects.

### Study Population

IGT subjects were consecutively recruited from those attending the CV prevention outpatient clinic of the Department of Internal Medicine, University of Parma. In subjects with impaired fasting plasma glucose an oral glucose tolerance test (OGTT) (75 g) was performed and IGT was defined according to criteria of the American Diabetes Association [23]. Inclusion criteria were IGT males or females aged 18–65 years. Exclusion criteria were stringent to exclude possible confounding factors and included the following: previous history of CVD, diabetes, medical history of neoplastic disease, acute or chronic illnesses, alcohol intake >80 g/day and chronic/intermittent medications. All participants had their medical history and anthropometric parameters recorded. Venous plasma samples (60 ml) were drawn for the determination of the main biochemical and metabolic parameters and for early EPC isolation. Five subjects (35.7%) refused to perform a second blood test for late-outgrowth EPC isolation.

**Figure 1:**
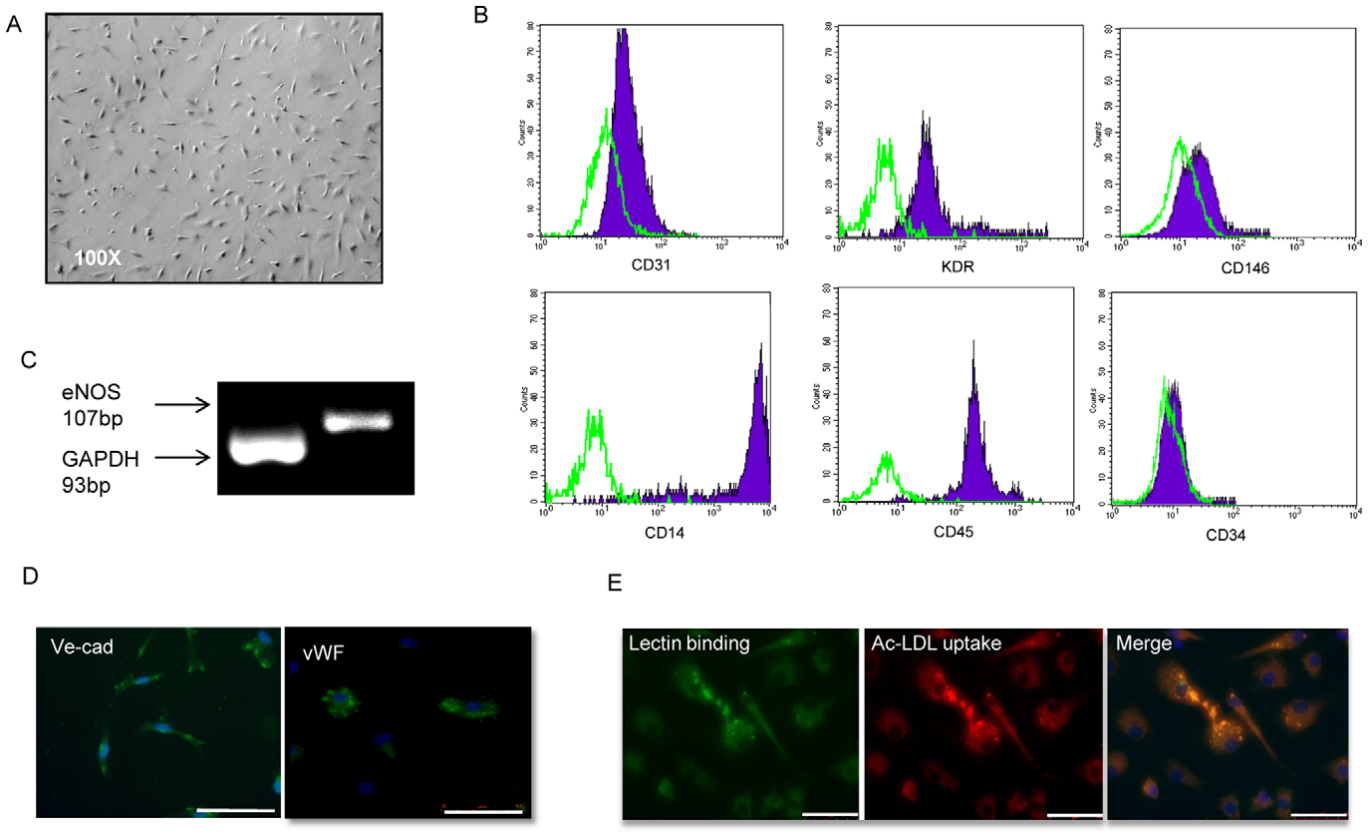
Characterization of early EPCs. Morphology and phenotype of early EPCs derived from human peripheral blood mononuclear cells. Cells were fibronectin-adherent with a spindle-shaped morphology (A). Representative flow cytometry analysis of cultured early EPCs for the expression of CD31, KDR (kinase insert domain receptor), CD146, CD14, CD45, and CD34. Plots show specific antibody staining (filled) versus isotype contro IgG staining (empty) (B). eNOS and GAPDH gene expression in early EPCs (C). Immunofluorescent staining of early EPCs for vWF (von Willebrand factor) and Ve-cad (vascular endothelial cadherin) (bar = 50 µm) (D) and of DiI-LDL (red) and fluorescein isothiocyanate-lectin (green) (bar = 50 µm) (E) (bp = base pairs; eNOS = endothelial nitric oxide synthase; GAPDH = Glyceraldehyde 3-phosphate dehydrogenase).

**Figure 2:**
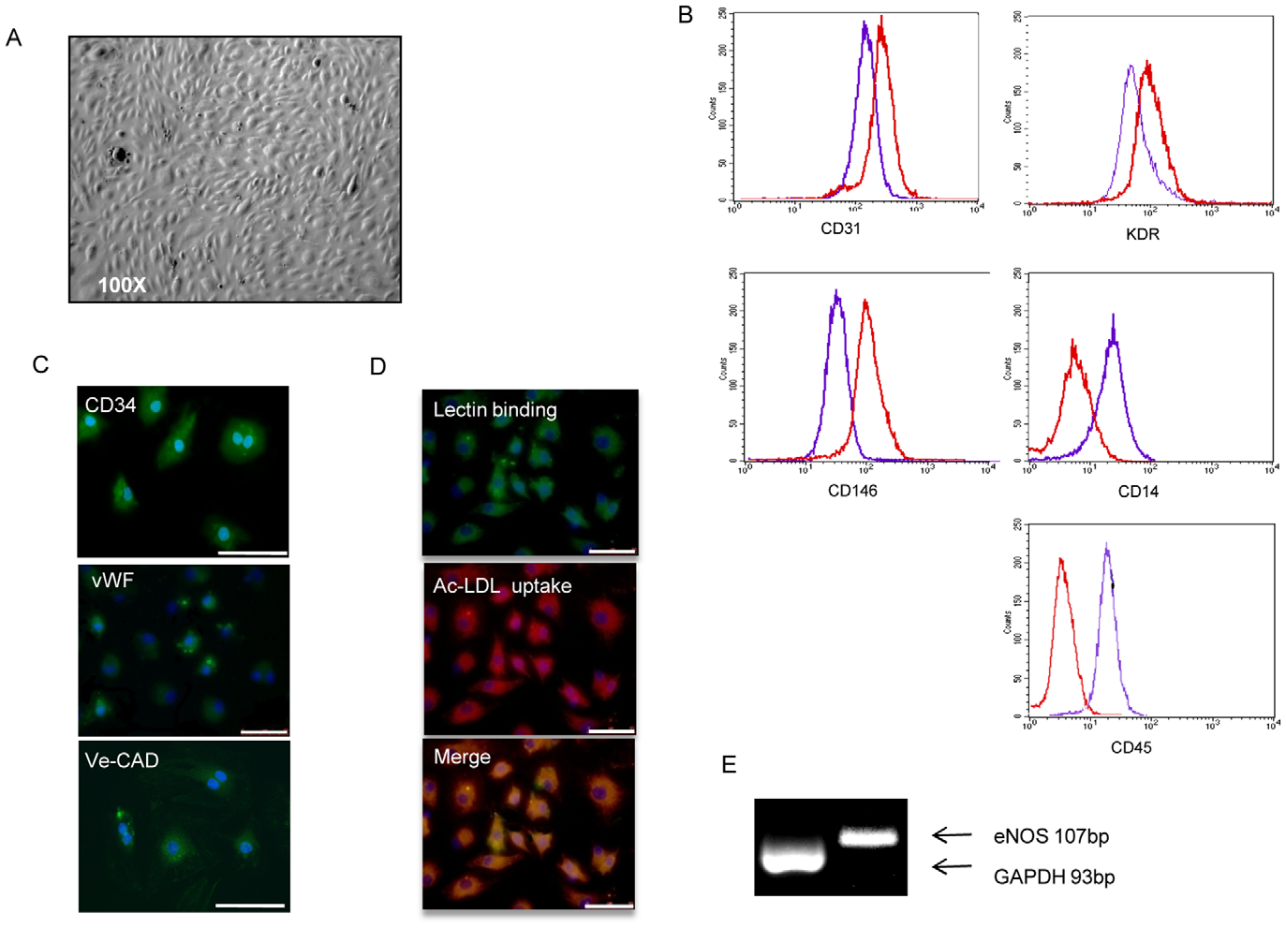
Characterization of late-outgrowth EPCs. Morphology and phenotype of late-outgrowth EPC derived from human peripheral blood mononuclear cells. Colonies of late-outgrowth EPCs with a cobblestone-like morphology (A). Representative flow cytometry analysis of late-outgrowth EPCs (red) compared to early EPCs (purple) for the expression of CD31, KDR, CD146 and CD14 (B). Immunofluorescent staining of late-outgrowth EPCs for CD34, Ve-cad (vascular endothelial cadherin) and vWF (von Willebrand factor) (C), immunofluorescence staining of late-outgrowth EPC for DiI-LDL (red), lectin (green) and merge (bar = 50 µm) (D). eNOS and GAPDH gene expression in late EPCs (E) (bp = base pairs; eNOS = endothelial nitric oxide synthase; GAPDH = Glyceraldehyde 3-phosphate dehydrogenase).

### Isolation and Culture of EPCs

EPC isolation was performed according to validated protocols [24], [25]. Peripheral blood mononuclear cells (PBMCs) were isolated by Lymphoprep (SENTINEL, Milan, Italy) density gradient centrifugation. PBMCs were cultured into six-well tissue fibronectin-coated (10 µM) culture plates at a density of 5×10^6^ cells/well, and grown in endothelial cell growth medium (EGM-2) (Lonza, Milano, Italy) supplemented with hEGF (human epidermal growth factor), hydrocortisone, gentamicin and amphotericin-B, 2% FBS (fetal bovine serum), VEGF (vascular endothelial growth factor), hFGF-B (human fibroblast growth factor), R3-IGF-1 (R3- insulin-like growth factor-1), ascorbic acid, heparin in 5% CO_2_ at 37°C. Culture medium was changed on day 5 and subsequently every 2 days. On day 7, adherent cells displaying an elongated spindle-shaped morphology were characterized to confirm early EPC/CAC phenotype [24]. For the isolation of late-outgrowth EPCs culture experiments were prolonged up to 3 weeks [24]. Late-outgrowth EPCs were isolated in a subgroup of 9/14subjects (64.3%). At day 21 adherent cells showed an endothelial-like morphology and were characterized to confirm the endothelial phenotype. The effect of pioglitazone was tested by culturing cells in three different conditions: 10 µM pioglitazone [21], [26] (kindly provided by Takeda Chemical, Osaka, Japan), in the presence or absence of 1 µM of the PPARγ-antagonist GW9662 (Sigma Aldrich, Milan, Italy), or vehicle (0.2% DMSO) as a control. Culture stimuli were added at the time of isolation (day 0) and then every time the medium was changed, up to the time cells were processed. All experiments were performed at day 7 and at day 21 for early and late-outgrowth EPCs, respectively.

**Figure 3:**
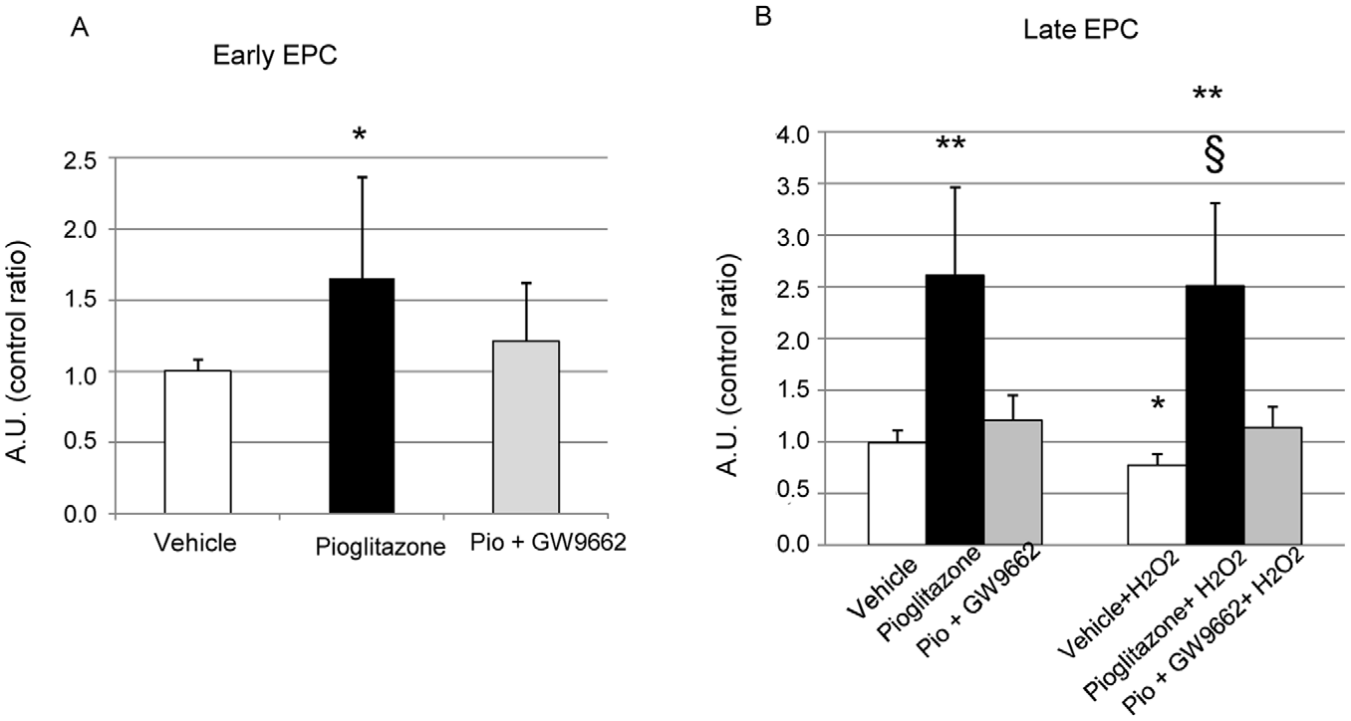
Effect of pioglitazone on EPC viability. The effects of pioglitazone (10 µM), pioglitazone (10 µM) + GW9662 (1 µM) and 0.2% DMSO vehicle culture conditions were examined on early (A) and late-outgrowth EPC viability alone and in the presence of H_2_O_2_ (500 µM, 24 h) (B); (*p<0.05 vs vehicle; **p<0.01 vs vehicle; §p<0.01 vs vehicle+H_2_O_2_).

### Characterization of Cultured EPCs by Flow Cytometry

In order to evaluate the phenotype of cultured EPCs, 5×10^5^ adherent cells were detached with trypsin-EDTA and analyzed by flow cytometry (FACS Calibur, BD Biosciences, Franklin Lakes, NJ) for the expression of CD45, CD14, CD31, CD146, CD34, CD3, CD19, CD64 (BD Biosciences) and KDR (kinase insert domain receptor) (R&D Systems, Minneapolis, MN, USA) surface markers and at least 2×10^4^ events were acquired for each analysis. Isotype Immunoglobulins (IgG1, IgG2) were used as controls (BD Biosciences). A single trained operator performed all flow cytometric test and analyses.

### Characterization of Cultured EPCs by Immunofluorescence

Fluorescent staining of adherent cells on 4-well chamber slides (Sigma Aldrich, Milan, Italy) was conducted to evaluate the expression of vWf (von Willebrand factor), VE-cad (vascular endothelial cadherin) and CD68 on early and late-outgrowth EPCs. Immunostaining with specific antibodies was performed. Cells were fixed with 4% paraformaldehyde for 30 min at room temperature and then blocked with 20% of rabbit/goat serum and incubated overnight at 4°C in the dark with anti-vWf (1∶50 Santa Cruz Biotechnology, Santa Cruz, CA, USA), anti-VE-cad (1∶50 Santa Cruz Biotechnology) and anti-CD68 (1∶50 Novus Biologicals, Littleton, CO, US) primary antibodies. After washing with PBS (phosphate buffered saline), cells were incubated with secondary antibody (1∶70) for 45 min at 37°C in the dark. Nuclei were counterstained with DAPI (4′,6-diamidine-2-phenyndole, Sigma Aldrich) and cover slips mounted with Vectashield (Vector Laboratories, Burlingame, CA, USA). Positive cells were then visualized under a fluorescent microscope (Olympus CX21,. Center Valley, PA, USA). Both early and late-outgrowth EPCs were also characterized for the uptake of 1,1′-dioctadecyl-3,3,3′,3′-tetramethylindocarbocyanine-labeled acetylated low-density lipoprotein (Dil-acLDL) (2.5 mg/ml: Molecular Probes, Eugene, OR, USA) and lectin binding. Briefly, after washing with PBS, cells were incubated for 3 h at 37°C with 1 µg/ml DiI–AcLDL. Cells were then fixed with 4% paraformaldehyde for 30 min and incubated for 2 hour with 10 mg/ml FITC-labeled *Ulex europaeus* agglutinin-1 lectin (UEA-1; Sigma Aldrich). After staining, samples were observed with an inverted fluorescent microscope (Leica. Microsystems GmbH, Wetzlar, Germany).

**Figure 4:**
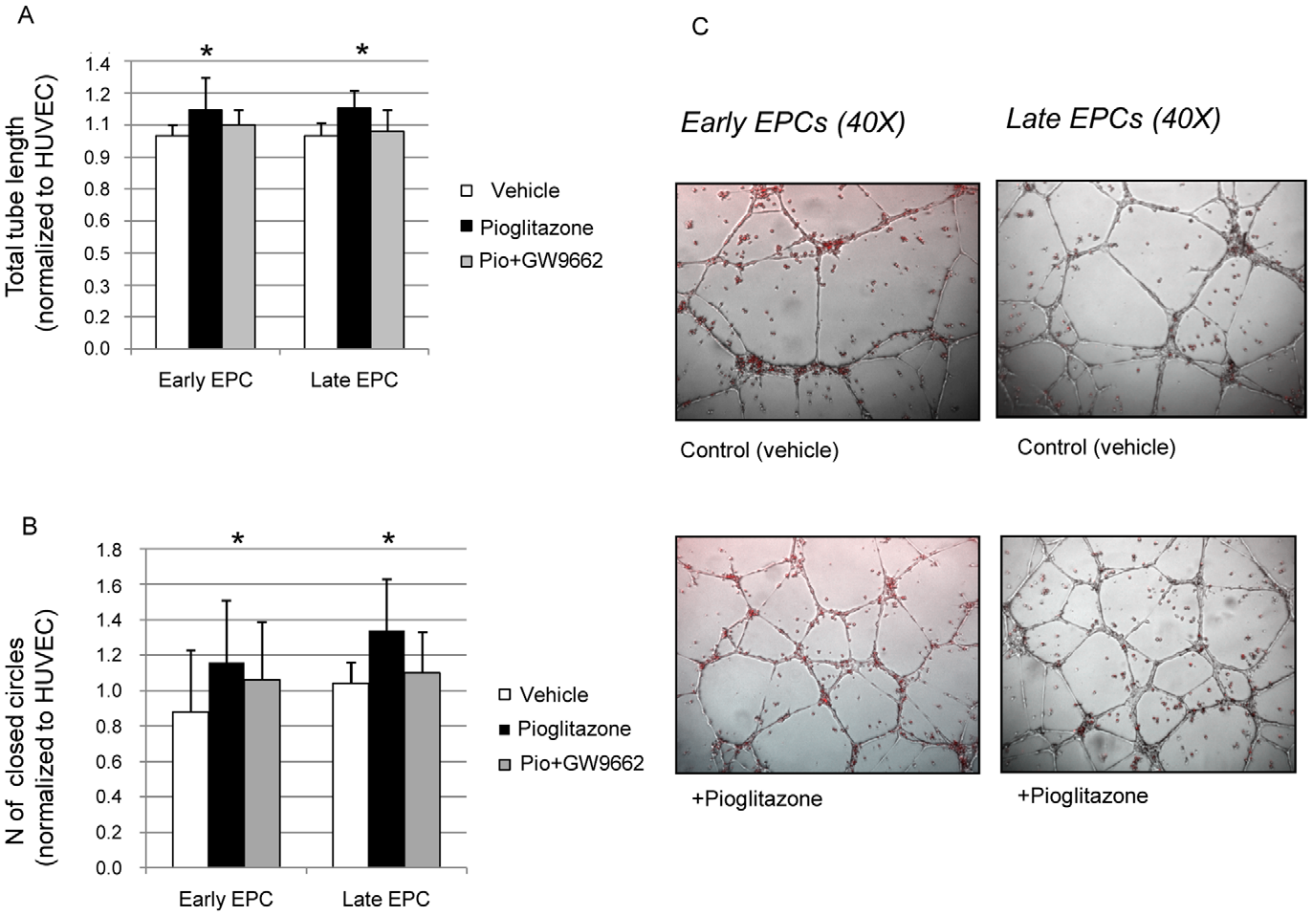
Effect of pioglitazone on EPC function. Effect of pioglitazone (10 µM), pioglitazone + GW9662 (1 µM) and vehicle culture conditions on early and late-outgrowth EPC tube formation capacity expressed as total tube length (A) and as number of closed circles formed by tube-like structures (B); representation of early and late-outgrowth EPC tube formation assay showing the network formed by EPCs plus HUVEC on Matrigel (EPCs are red stained with Dil) in the presence of pioglitazone and vehicle (C); (*p<0.05 vs vehicle) (Pio = pioglitazone).

### Apoptosis Assay

Early stages of apoptosis in EPCs were investigated with Annexin V-FITC Apoptosis Detection Kit Plus (BioVision, Mountain View, CA). Early and late-outgrowth EPCs were harvested by trypsinization and 5×10^5^ cells were stained with Annexin-V-FITC and propidium iodide in binding buffer for 10 min at room temperature following manufacturer's instructions. Stained cells were analysed by flow cytometry (FACS Calibur, BD) using CellQuest software (Becton Dickinson) by a single blinded operator.

**Figure 5:**
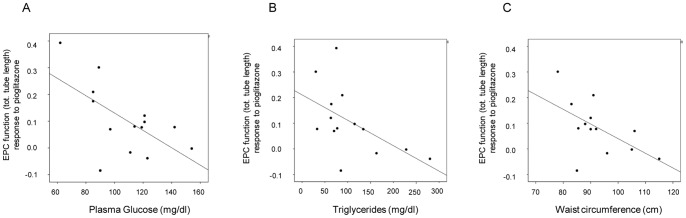
Association between EPC function response to pioglitazone and clinical characteristics. Pioglitazone-induced EPC function was negatively associated with fasting plasma glucose (r = −0.61; p = 0.020) (A), triglycerides (r = −0.55; p = 0.044)(B) and waist circumference (r = −0.56; p = 0.045) (C).

### Viability Assay

Cell viability was evaluated by VisionBlue fluorescence cell viability assay kit (Biovision) following manufacturer's instructions. At day 0, PBMCs were seeded on fibronectin-coated 96-well tissue culture plates at a density of 5×10^5^ cells/well in a volume of 200 µl of EGM-2 and cultured to obtain early and late-outgrowth EPCs in the three culture conditions as previously described. After washing, adherent cells were resuspended in 100 µl EGM-2 plus 10 µl VisionBlue reagent followed by incubation for 2 hours at 37°C. The fluorescent product was measured in a fluorescent plate reader (excitation: 540 nm, emission: 586 nm) Cary Eclipse fluorescence spectrophotometer (Varian/Agilent, Santa Clara, CA, USA). To test the effect of pioglitazone on EPC viability in a oxidative stress condition, H_2_O_2_ (500 µM, 24 h) was added to late-outgrowth EPC culture medium. Data were normalized for vehicle control values.

**Figure 6:**
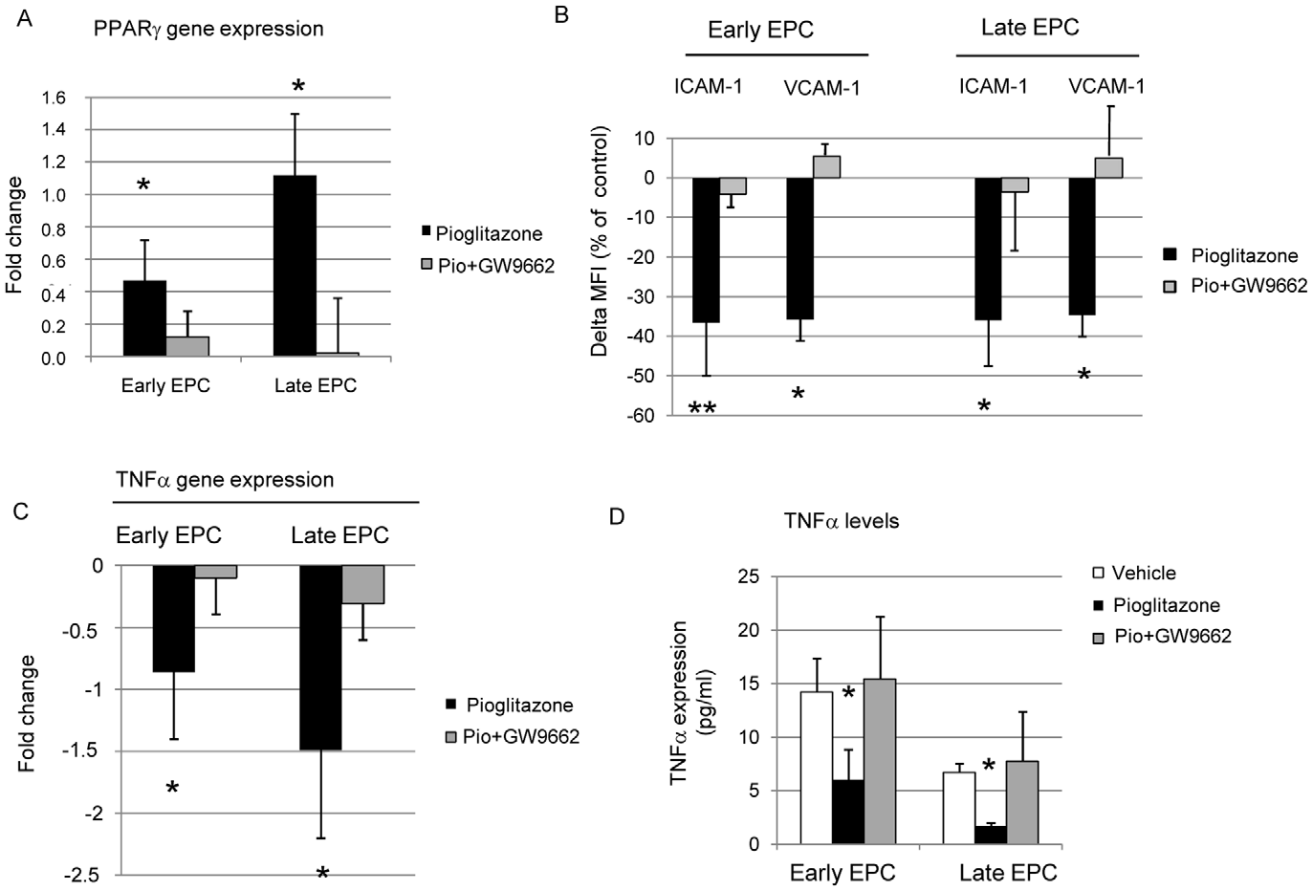
Pioglitazone effect on EPC pro-inflammatory molecule expression. Effect of pioglitazone on PPARγ, adhesion molecule and TNFα expression in EPCs. PPARγ gene expression in early and late-outgrowth EPC exposed to pioglitazone (10 µM) and pioglitazone + GW9662 (1 µM) (A) Effect of pioglitazone in modulating ICAM-1 and VCAM-1 expression by early and late-outgrowth EPCs (cytofluorimetric analyses). Results are reported as delta % of MFI (mean fluorescence intensity) with vehicle condition (B). Effects of pioglitazone on TNFα gene (C) and protein (D) expression in early and late-outgrowth EPC. (*p<0.05 vs vehicle; **p<0.01 vs vehicle). Real time PCR data are expressed as −ΔΔCt and represent the relative gene expression of EPC cultured in the presence of pioglitazone 10 µM (with or without GW9662 1 µM) in relation to vehicle, normalized for the endogenous control GAPDH. Protein expression is measured with ELISA assay from culture supernatants. Results from five independent experiments performed in duplicate are shown (*p<0.05 vs vehicle) (Pio = pioglitazone).

### EPC Function (Tube Formation Assay)

The capacity of early and late-outgrowth EPCs to cooperate to tubular-like structure formation when co-cultured with mature endothelial cells, was examined in Matrigel culture (BD Biosciences) [27]. Briefly, 50 µl of Matrigel (BD Biosciences) was added to prechilled 96-well plates. Matrigel was allowed to polymerize for 30 min at 37°C. Human umbilical vein endothelial cells (HUVECs) (2×10^4^), were cultured in triplicate in EGM-2 medium on Matrigel with or without the addition of EPCs (3×10^3^) obtained in the different culture conditions. After 24 hours, the number of closed circles and sprouts formed by tubular-like structures, total tube length, mean tube length and number of tubes were counted in each well. The capacity of EPCs to participate to tube formation was expressed as the ratio of tube formation assay measurements in the culture of HUVECs plus EPCs to the mean values of tube formation assay measurements in the culture of HUVECs alone. Each conditions was tested in triplicate and intra-individual coefficient of variation of total tube length was 4.46±2.80% and 3.82±1.56% for early and late-outgrowth EPCs respectively.

### EPC Pro-inflammatory Molecule Assessment

EPC inflammatory response to pioglitazone was investigated in early and late-outgrowth EPCs isolated from healthy donor buffy coats. PBMCs were isolated and EPCs were cultured in the three different conditions as described in the dedicated paragraph. Measurements were performed in 5 independent experiments.

#### Expression of adhesion molecules in EPCs

Adhesion molecule expression was assessed in 5×10^5^ early and late-outgrowth EPCs detached with trypsin-EDTA and analyzed by flow cytometry (FACS Calibur, BD Biosciences) for the expression of intercellular adhesion molecule 1 (ICAM-1) and vascular cell adhesion molecule 1 (VCAM-1) (BD Bioscences).

#### eNOS, PPARγ and pro-inflammatory cytokine/chemokine gene expression in EPCs

Early and late-outgrowth EPCs were lysed with QIAzol lysis reagent (Qiagen Ltd, West Sussex, UK) and stocked at −80°C. Total RNA was extracted using miRNeasy Mini Kit (Qiagen) and quantified by NanoDrop (NanoDrop Technologies, DE). The cDNA was obtained using high capacity RNA-to-cDNA Kit (Applied Biosystems, Carlsbad, CA, USA) starting from 250 ng of total RNA and in accordance with manufacturer's instructions. Reactions without reverse transcriptase were carried out in parallel as a negative control. Quantitative RT-PCR was performed to determine the expression of eNOS, PPARγ, interleukin(IL)-6, IL-8, tumor necrosis factor α (TNFα), and monocyte chemoattractant protein-1 (MCP-1) together with the transcription factor nuclear factor kappa-light-chain-enhancer of activated B cells (NF-kB). Gene expression measurements were evaluated using TaqMan Gene expression Master Mix (Applied Biosystems) with TaqMan primers and probe (Applied Biosystems) on a StepOne Real-Time PCR system (Applied Biosystem). All samples were analyzed in triplicate and the mean value was considered. Thermal cycling conditions were as follows: 50°C for 2 min, 95°C for 10 min, followed by 40 amplification cycles (95°C for 15 s; 60°C for 1 min). Gene expression values were calculated based on the ΔΔCt method [28], resulting in a relative level of expression. The relative level of mRNA expression was calculated using human GAPDH (glyceraldehyde 3-phosphadehydrogenase).

#### ELISA assay of pro-inflammatory cytokine/chemokines

Quantification of the levels of cytokines IL-6 and TNFα and chemokines IL-8 and MCP-1, was performed on culture supernatants using a commercially available high-sensitivity multiplexed enzyme-linked immunosorbent assay kit (SearchLight Multiplex Immunoassay Kits, Aushon) according to manufacturer's instructions (Aushon Biosystems, Billerica, MA, USA). Cytokine/chemokine concentrations in EPC supernatants were evaluated by establishing a standard curve with serial dilutions of the recombinant human proteins. The image was detected using SearchLight Plus Imaging and Analysis System and analyzed with the Searchlight Array Analyst software (Aushon Biosystems). Values from ELISA assays were normalized to total cell number.

#### NF-kB activation assay

NF-kB activation was assessed with a sensitive multi-well colorimetric assay for nuclear NF-kB (p50) (TRANS-AM; Active Motif, Rixensart, Belgium) [29] following manufacturer's instructions.

### Statistical Analysis

Continuous normally distributed variables are expressed as mean±SD; skewed variables are reported as median (25^th^–75^th^ interquartiles). One-way repeated measures ANOVA followed by Bonferroni post-hoc test or Student's unpaired *t* test were used to compare different experimental conditions. Early EPC function response to pioglitazone was expressed as [total tube length_(pio)_ –total tube length_(vehicle)_]/total tube length_(vehicle)_. Statistical significance was accepted at p<0.05. All analyses were performed using a Windows-based SPSS statistical package (Version 19.0, Chicago, IL, USA).

## Results

### Characteristics of the Study Population

The study population included 7 male and 7 female IGT subjects (mean age was 58±6 years). Clinical, metabolic and biochemical variables are reported in Table 1.

### Early and Late-outgrowth EPC Characterization

Early EPCs exhibited a spindle-like shape (Figure 1A) and displayed a range of endothelial cell-surface markers, including KDR (92%) CD31 (75%), CD146 (18%) (Figure 1B) and vWF (Figure 1C). Consistent with previous reports [30], these cells also expressed leukocyte cell-surface markers such as CD45 (99%), and CD14 (98%) (Figure 1B). Macrophage marker CD68 expression resulted of 16% by immunofluorescence and that of the stem cell marker CD34 very low (2%). Early EPCs were double positive for Ac-LDL uptake and lectin binding properties (Figure 1C). According to literature [25], late-outgrowth EPCs were able to form rare colonies of cells with a cobblestone-like morphology (Figure 2A). Late-outgrowth EPCs expressed endothelial markers VE-cad and vWF together with CD34 (Figure 2C), as shown by immunofluorescence. Compared to early EPCs, late EPCs showed a higher expression of the endothelial markers CD31 (98%), KDR (96%) and CD146 (92%) (Figure 2B), whereas that of the monocyte antigen CD14, CD45 (Figure 2B) and CD68 was negative. Late-outgrowth EPCs were also double positive for Ac-LDL uptake and lectin binding capacities (Figure 2D). Lymphocyte contamination was excluded by the absence of CD3 and CD19 expression in early and late-outgrowth EPCs. eNOS gene expression was observed in both EPC populations (Figure 1C and 2E).

### Effect of Pioglitazone on EPC Apoptosis and Viability

The addition of pioglitazone to culture medium was not associated with changes in rate of apoptosis in either early or late-outgrowth EPCs (data not shown).

Pioglitazone improved early and late-outgrowth EPC viability by 65% (95% CI: 23–107%, p = 0.016, Figure 3A). and 161% (95% CI: 37–285%, p = 0.009, Figure 3B) respectively, compared to vehicle In the presence of 500 µM H_2_O_2_, viability of late-outgrowth EPCs was reduced by 23% (95% CI: 2–45%, p = 0.030); this effect was prevented when pioglitazone was combined with H_2_O_2_ (p = 0.005 vs vehicle, Figure 3B). The effect of pioglitazone on EPC viability was PPARγ-mediated since the effect was blocked by GW9662.

### Effect of Pioglitazone on EPC Function

The capacity of EPCs to form tubular structures is one of the most important functional properties of EPCs [27]. The addition of pioglitazone improved the capacity of EPCs to form tubular-like structures (Figure 4C) expressed as: number of closed circles formed by early (mean increase of 28%, p = 0.005) and late-outgrowth (mean increase of 30%; p = 0.037) EPCs (Figure 4B); total tube length in early (mean increase of 11%; p = 0.026) and late-outgrowth (mean increase of 12%, p = 0.031) EPCs (Figure 4A); tube number in early (mean increase of 12%; p = 0.001) and late-outgrowth (mean increase of 23%; p = 0.007) EPCs; number of sprouts in early (mean increase of 20%; p = 0.001) and late-outgrowth (mean increase of 31%; p = 0.011) EPCs compared to control. The observed effects were PPARγ mediated, since the addition of GW9662 together with pioglitazone did not significantly improve EPC function.

### Association between EPC Function Response to Pioglitazone and Clinical Characteristics

Early EPC function response to pioglitazone was found to be inversely correlated with fasting plasma glucose (r = −0.61; p = 0.020), triglycerides (r = −0.55; p = 0.044) and waist circumference (r = −0.56; p = 0.045) (Figure 5A–C).

### Effects of Pioglitazone on PPARγ Gene Expression

PPARγ mRNA was expressed in both early and late-outgrowth EPC. The addition of pioglitazone significantly (p<0.05) increased PPARγ expression in both cell populations and was prevented by the addition of GW9662 (Figure 6A).

### EPC Pro-inflammatory Profile

#### Effect of pioglitazone on adhesion molecule expression

Pioglitazone significantly reduced ICAM-1 expression in both early (−36.5±4.14%;p = 0.001) and late-outgrowth (−35.9±11.6%, p = 0.012) EPCs. Likewise, VCAM-1 expression was also significantly decreased by pioglitazone in both early (−35.7±5.3%; p = 0.047) and late-outgrowth (−35.6±5.4%; p = 0.048) EPCs compared to vehicle. These results were also shown to be PPARγ-mediated, since the addition of GW9662 prevented these effects (Figure 6B).

#### Effect of pioglitazone on inflammatory cytokine/chemokine expression

The presence of pioglitazone significantly decreased TNFα gene expression, measured by RT-PCR in both early (p = 0.034) and late-outgrowth (p = 0.026) EPCs (Figure 6C). TNFα levels measured by ELISA paralleleled the mRNA expression findings as in both early (p = 0.022) and in late-outgrowth (p = 0.017) EPCs (Figure 6D). The levels of IL-6, IL-8 and MCP-1 gene and protein expression remained unchanged in cells following treatment with pioglitazone (data not shown).

#### Effect of pioglitazone on NF-kB gene expression and activation

Gene expression assay showed no difference in NF-kB gene expression in early (p = 0.31) or late-outgrowth (p = 0.19) EPCs in the presence of pioglitazone. Similar findings were observed in NF-kB activation assay showing no differences between vehicle and pioglitazone-cultured early (p = 0.81) or late-outgrowth (p = 0.78) EPCs (data not shown).

## Discussion

The main finding of this pre-clinical study is the demonstration of a direct *in vitro* favourable vascular effect of pioglitazone. The addition of pioglitazone *in vitro* improved viability and capacity to form tubular-like structures, in early and late-outgrowth EPCs obtained from IGT subjects. Furthermore, pioglitazone showed a potential anti-inflammatory action in reducing the levels of EPC adhesion molecules and TNFα expression. These observed effects were PPARγ-specific, since they were prevented by the addition of the PPARγ-antagonist.

Recent studies have demonstrated that pioglitazone treatment improves plaque stability and endothelial function [5]–[7] in IGT patients, suggesting that its beneficial effects may also occur in pre-diabetic conditions. Experiments conducted in cultured EPCs supported a positive action of pioglitazone on key regulators of atherosclerosis independently of its metabolic action [31], [32]. Our study highlights an additional putative mechanism whereby pioglitazone therapy may reduce CV risk in IGT individuals, independent of the insulin-sensitising action.

In the present study the effects of pioglitazone were examined in two distinct EPC populations isolated *ex vivo* retaining a complementary function in vascular repairing mechanisms [19]. It is recognised that early EPCs show a leukocyte origin [30], [33], and have also been referred to as circulating angiogenic cells (CACs) since they secrete regulators of angiogenesis [30] and appear to promote neovascularization in animal models of hind-limb ischemia [34] and myocardial infarction [35]. Late-outgrowth EPCs retain the ability to proliferate and showed a high vascular regenerative potential [36], [37]. Given the different features and origins of these two EPC populations, the evaluation of pioglitazone effects on both phenotypes serves to provide a more complete insight into EPC biology.

Pioglitazone treatment has been shown to increase the number of circulating EPCs and prevent apoptosis in wild-type mice in a phosphatidylinositol 3-kinase-dependent manner [38]. Furthermore, pioglitazone treatment increased EPC levels in diabetic patients [20] and in subjects with CAD and normal glucose tolerance [21].

The main finding of our study is that pioglitazone improved early and late-outgrowth EPC function, as measured by EPC capacity to participate to tubular-like structure formation *in vitro*. These results extend previous observations where pioglitazone treatment ameliorated EPC functional properties in terms of migratory response in both animal and human studies. Pioglitazone increased migratory response and adhesion capacity in type 2 diabetic subjects [20]. Similarly, EPCs cultured from mice treated with pioglitazone have shown a higher migratory capacity compared to placebo control [38]. Analogous effects in EPC migration were observed in subjects with CAD and normal glucose tolerance treated with pioglitazone for 30 days [21]. Studies have attempted to investigate possible mechanisms to explain the observed effects of pioglitazone on EPC function [39]. The synthesis of nitric oxide via endothelial cells has been shown to be a key regulator of EPC release [40]; however, this hypothesis has not been confirmed by others [38]. Other evidence has shown that pioglitazone treatment reduces basal and stimulated NADPH oxidase activity with consequent reduction in oxidative stress and EPC apoptosis [21]. In agreement with this observation and other studies [20], [38], we observed that pioglitazone prevented H_2_O_2_-induced cell death in late-outgrowth EPCs, suggesting a protective role of this drug against oxidative stress-induced cell death.

Pioglitazone anti-inflammatory effects, in terms of a reduction in pro-inflammatory cytokine and adhesion molecule expression in mature stimulated endothelial cells [22], [41], [42], were also demonstrated for the first time in our study in unstimulated early and late-outgrowth EPCs. However, pioglitazone did not modify other inflammatory cytokines nor NF-kB gene expression and activity. This is in contrast with previous reports indicating that anti-inflammatory effects of pioglitazone are sustained by a transrepression mechanism by which the activated PPARγ interferes with the activity of pro-inflammatory transcription factors, such as NF-kB [43], [44]. However, the hypothesis that the receptor can directly interact with other signaling proteins such as ERK [45], MAPK [46] and PKC1a [47], has also been described.

Future studies are required to unravel the precise mechanisms by which these anti-inflammatory effects in EPCs are exerted.

Unexpectedly, in our study EPC function response to pioglitazone was inversely correlated with some clinical parameters in IGT subjects. This finding is partially supported by the work by Werner *et al.* [21] showing that pioglitazone exerted beneficial effects on EPC number and migratory capacity in patients with normal glucose tolerance, supporting the idea that this agent may also be beneficial for normoglycemic individuals when presenting a higher CV risk.

The present study presents some limitations. The lack of a control group did not allow us to observe any possible alteration in the function of EPC isolated from IGT subjects, as already demonstrated in the presence of diabetes [20]. The sample size was also lower than desired, which precluded the possibility of subgroup analysis and assessment of associations between multiple parameters. Although we demonstrated a role of pioglitazone in reducing EPC pro-inflammatory molecule expression, the precise molecular mechanisms mediating pioglitazone effects on EPC biology remain unclear and still need to be addressed. The inflammatory response was evaluated in unstimulated conditions in contrast with other studies in which inflammation was assessed following diverse stimuli (i.e. LPS) in which pioglitazone showed a significant reduction of TNFα expression levels in mononuclear cells [48] and in adypocytes [49] cultured *ex vivo*. This approach would have been useful to confirm and strengthen our findings.

In conclusion, EPCs have clearly emerged as a new dimension in vascular biology. In patients with metabolic alterations such as compensatory hyperinsulinemia, impaired fasting glucose and IGT, EPC number and function are impaired. This preclinical study shows the *in vitro* effect of pioglitazone in ameliorating EPC angiogenic capacity and inflammation. Improvement in EPC function may represent a clinically relevant effect of pioglitazone that could potentially benefit patients with vascular disease before the progression to overt diabetes mellitus. In this study we have shown for the first time that pioglitazone exerts beneficial *in vitro* effects on EPCs isolated from IGT subjects, supporting a novel clinical therapeutic implication of pioglitazone in reducing/preventing CV risk in pre-diabetic states, although potential clinical effects need to be confirmed in human randomized studies.

## References

[1] Mazzone T, Meyer PM, Feinstein SB, Davidson MH, Kondos GT, et al (2006) Effect of pioglitazone compared with glimepiride on carotid intima-media JAMA. 296: 2572–81. 10.1001/jama.296.21.joc60158 17101640

[2] Dormandy JA, Charbonnel B, Eckland DJ, Erdmann E, Massi-Benedetti M, et al (2005) PROactive investigators. Secondary prevention of macrovascular events in patients with type 2 diabetes in the PROactive Study (PROspective pioglitAzone Clinical Trial In macroVascular Events): a randomised controlled trial. Lancet 366: 1279–89. 16214598 10.1016/S0140-6736(05)67528-9

[3] Fuller JH, Shipley MJ, Rose G, Jarrett RJ, Keen H (1980) Coronary-heart-disease risk and impaired glucose tolerance. The Whitehall study. Lancet 1: 1373–6. 6104171 10.1016/s0140-6736(80)92651-3

[4] Mizoguchi M, Tahara N, Tahara A, Nitta Y, Kodama N, et al (2011) Pioglitazone attenuates atherosclerotic plaque inflammation in patients with impaired glucose tolerance or diabetes a prospective, randomized, comparator-controlled study using serial FDG PET/CT imaging study of carotid artery and ascending aorta. JACC Cardiovasc Imaging 4: 1110–8. 21999871 10.1016/j.jcmg.2011.08.007

[5] Yang HB, Zhao XY, Zhang JY, Du YY, Wang XF Pioglitazone induces regression and stabilization of coronary atherosclerotic plaques in patients with impaired glucose tolerance. Diabet Med. (in press). 10.1111/j.1464-5491.2011.03458.x 21950726

[6] Nakayama T, Komiyama N, Yokoyama M, Namikawa S, Kuroda N, et al (2010) Pioglitazone induces regression of coronary atherosclerotic plaques in patients with type 2 diabetes mellitus or impaired glucose tolerance: a randomized prospective study using intravascular ultrasound. Int J Cardiol 138: 157–65. 18817993 10.1016/j.ijcard.2008.08.031

[7] Quinn CE, Lockhart CJ, Hamilton PK, Loughrey CM, McVeigh GE (2010) Effect of pioglitazone on endothelial function in impaired glucose tolerance. Diabetes Obes Metab 12: 709–15. 20590748 10.1111/j.1463-1326.2010.01224.x

[8] Duan SZ, Usher MG, Mortensen RM (2008) Peroxisome proliferator-activated receptor-gamma-mediated effects in the vasculature. Circ Res 102: 283–94. 18276926 10.1161/CIRCRESAHA.107.164384

[9] Brown JD, Plutzky J (2007) Peroxisome proliferator-activated receptors as transcriptional nodal points and therapeutic targets. Circulation 115: 518–33. 17261671 10.1161/CIRCULATIONAHA.104.475673

[10] Asahara T, Murohara T, Sullivan A, Silver M, van der Zee R, et al (1997) Isolation of putative progenitor endothelial cells for angiogenesis Science. 275: 964–7. 10.1126/science.275.5302.964 9020076

[11] Kirton JP, Xu Q (2010) Endothelial precursors in vascular repair. Microvasc Res 79: 193–9. 20184904 10.1016/j.mvr.2010.02.009

[12] Vasa M, Fichtlscherer S, Aicher A, Adler K, Urbich C, et al (2001) Number and migratory activity of circulating endothelial progenitor cells inversely correlate with risk factors for coronary artery disease. Circ Res 89: E1–E7. 11440984 10.1161/hh1301.093953

[13] Hill JM, Zalos G, Halcox JP, Schenke WH, Waclawiw MA, et al (2003) Circulating endothelial progenitor cells, vascular function, and cardiovascular risk. N Engl J Med 348: 593–600. 12584367 10.1056/NEJMoa022287

[14] Tepper OM, Galiano RD, Capla JM, Kalka C, Gagne PJ, et al (2002) Human endothelial progenitor cells from type II diabetics exhibit impaired proliferation, adhesion, and incorporation into vascular structures. Circulation 106: 2781–2786. 12451003 10.1161/01.cir.0000039526.42991.93

[15] Fadini GP, de Kreutzenberg SV, Coracina A, Baesso I, Agostini C, et al (2006) Circulating CD34+ cells, metabolic syndrome, and cardiovascular risk. Eur.Heart J 27: 2247–2255. 16912055 10.1093/eurheartj/ehl198

[16] Dei Cas A, Spigoni V, Ardigò D, Pedrazzi G, Franzini L, et al (2011) Reduced circulating endothelial progenitor cell number in healthy young adult hyperinsulinemic men. Nutr Metab Cardiovasc Dis 21: 512–7. 20227256 10.1016/j.numecd.2009.11.011

[17] Fadini GP, Pucci L, Vanacore R, Baesso I, Penno G, et al (2007) Glucose tolerance is negatively associated with circulating progenitor cell levels. Diabetologia 50: 2156–63. 17579827 10.1007/s00125-007-0732-y

[18] Rohde E, Malischnik C, Thaler D, Maierhofer T, Linkesch W, et al (2006) Blood monocytes mimic endothelial progenitor cells. Stem Cells 24: 357–367. 16141361 10.1634/stemcells.2005-0072

[19] Hur J, Yoon CH, Kim HS, Choi JH, Kang HJ, et al (2004) Characterization of two types of endothelial progenitor cells and their different contributions to neovasculogenesis. Arterioscler Thromb Vasc Biol 24: 288–293. 14699017 10.1161/01.ATV.0000114236.77009.06

[20] Wang CH, Ting MK, Verma S, Kuo LT, Yang NI, et al (2006) Pioglitazone increases the numbers and improves the functional capacity of endothelial progenitor cells in patients with diabetes mellitus. Am Heart J 152: 1051.e1–8. 10.1016/j.ahj.2006.07.029 17161050

[21] Werner C, Kamani CH, Gensch C, Böhm M, Laufs U (2007) The peroxisome proliferator-activated receptor-gamma agonist pioglitazone increases number and function of endothelial progenitor cells in patients with coronary artery disease and normal glucose tolerance. Diabetes 56: 2609–15. 17623816 10.2337/db07-0069

[22] Pasceri V, Wu HD, Willerson JT, Yeh ET (2000) Modulation of vascular inflammation in vitro and in vivo by peroxisome proliferator-activated receptor-gamma activators. Circulation 101: 235–8. 10645917 10.1161/01.cir.101.3.235

[23] Akhter J (1997) The American Diabetes Association's Clinical Practice Recommendations and the developing world. Diabetes Care 20: 1044–1045. 10.2337/diacare.20.6.1044b 9167126

[24] Fadini GP, Losordo D, Dimmeler S (2012) Critical reevaluation of endothelial progenitor cell phenotypes for therapeutic and diagnostic use. Circ Res 110(4): 624–37. 22343557 10.1161/CIRCRESAHA.111.243386 PMC3382070

[25] Prater DN, Case J, Ingram DA, Yoder MC (2007) Working hypothesis to redefine endothelial progenitor cells. Leukemia 21: 1141–9. 17392816 10.1038/sj.leu.2404676

[26] Majithiya JB, Paramar AN, Balaraman R (2005) Pioglitazone, a PPARgamma agonist, restores endothelial function in aorta of streptozotocin-induced diabetic rats. Cardiovasc Res 66: 150–61. 15769458 10.1016/j.cardiores.2004.12.025

[27] Arnaoutova I, George J, Kleinman HK, Benton G (2009) The endothelial cell tube formation assay on basement membrane turns 20: state of the science and the art. Angiogenesis 12: 267–74. 19399631 10.1007/s10456-009-9146-4

[28] Livak KJ, Schmittgen TD (2001) Analysis of relative gene expression data using real-time quantitative PCR and the 2(-Delta Delta C(T)) Method. Methods 25: 402–8. 11846609 10.1006/meth.2001.1262

[29] Renard P, Ernest I, Houbion A, Art M, Le Calvez H, et al (2001) Development of a sensitive multi-well colorimetric assay for active NFkappaB. Nucleic Acids Res 29: E21. 11160941 10.1093/nar/29.4.e21 PMC29628

[30] Rehman J, Li J, Orschell CM, March KL (2003) Peripheral blood “endothelial progenitor cells” are derived from monocyte/macrophages and secrete angiogenic growth factors. Circulation 107: 1164–9. 12615796 10.1161/01.cir.0000058702.69484.a0

[31] Walcher D, Marx N (2004) Insulin resistance and cardiovascular disease: the role of PPARgamma activators beyond their anti-diabetic action. Diab Vasc Dis Res 1: 76–81. 16302645 10.3132/dvdr.2004.011

[32] Redondo S, Hristov M, Gümbel D, Tejerina T, Weber C (2007) Biphasic effect of pioglitazone on isolated human endothelial progenitor cells: involvement of peroxisome proliferator-activated receptor-gamma and transforming growth factor-beta1. Thromb Haemost 97: 979–87. 17549301

[33] Medina RJ, O'Neill CL, Sweeney M, Guduric-Fuchs J, Gardiner TA, et al (2010) Molecular analysis of endothelial progenitor cell (EPC) subtypes reveals two distinct cell populations with different identities. BMC Med Genomics 3: 18. 20465783 10.1186/1755-8794-3-18 PMC2881111

[34] Kalka C, Masuda H, Takahashi T, Kalka-Moll WM, Silver M, et al (2000) Transplantation of ex vivo expanded endothelial progenitor cells for therapeutic neovascularization. Proc Natl Acad Sci U S A. 97: 3422–7. 10.1073/pnas.070046397 PMC16255 10725398

[35] Kawamoto A, Gwon HC, Iwaguro H, Yamaguchi JI, Uchida S, et al (2001) Therapeutic potential of ex vivo expanded endothelial progenitor cells for myocardial ischemia. Circulation 103: 634–7. 11156872 10.1161/01.cir.103.5.634

[36] Yoder MC, Mead LE, Prater D, Krier TR, Mroueh KN, et al (2007) Re-defining endothelial progenitor cells via clonal analysis and hematopoietic stem/progenitor cell principals. Blood 109: 1801–9. 17053059 10.1182/blood-2006-08-043471 PMC1801067

[37] Ingram DA, Mead LE, Moore DB, Woodard W, Fenoglio A, et al (2005) Vessel wall-derived endothelial cells rapidly proliferate because they contain a complete hierarchy of endothelial progenitor cells. Blood 105: 2783–6. 15585655 10.1182/blood-2004-08-3057

[38] Gensch C, Clever YP, Werner C, Hanhoun M, Böhm M, et al (2007) The PPAR-gamma agonist pioglitazone increases neoangiogenesis and prevents apoptosis of endothelial progenitor cells. Atherosclerosis 192: 67–74. 16876172 10.1016/j.atherosclerosis.2006.06.026

[39] Schernthaner G (2009) Pleiotropic effects of thiazolidinediones on traditional and non-traditional atherosclerotic risk factors. Int J Clin Pract 63: 912–29. 19490202 10.1111/j.1742-1241.2009.02025.x

[40] Aicher A, Heeschen C, Mildner-Rihm C, Urbich C, Ihling C, et al (2003) Essential role of endothelial nitric oxide synthase for mobilization of stem and progenitor cells. Nat Med 9: 1370–6. 14556003 10.1038/nm948

[41] Marx N, Walcher D (2007) Vascular effects of PPARgamma activators - from bench to bedside. Prog Lipid Res 46: 283–96. 17637478 10.1016/j.plipres.2007.05.003

[42] Ishibashi M, Egashira K, Hiasa K, Inoue S, Ni W, et al (2002) Antiinflammatory and antiarteriosclerotic effects of pioglitazone. Hypertension 40: 687–93. 12411463 10.1161/01.hyp.0000036396.64769.c2

[43] Yuan Z, Liu Y, Liu Y, Zhang J, Kishimoto C, et al (2005) Cardioprotective effects of peroxisome proliferator activated receptor gamma activators on acute myocarditis: anti-inflammatory actions associated with nuclear factor kappaB blockade. Heart 9: 1203–8. 10.1136/hrt.2004.046292 PMC1769084 15774612

[44] Ao C, Huo Y, Qi L, Xiong Z, Xue L, et al (2010) Pioglitazone suppresses the lipopolysaccharide-induced production of inflammatory factors in mouse macrophages by inactivating NF-kappaB. Cell Biol Int 34: 723–30. 19947950 10.1042/CBI20090005

[45] Burgermeister E, Chuderland D, Hanoch T, Meyer M, Liscovitch M, et al (2007) Interaction with MEK causes nuclear export and downregulation of peroxisome proliferator-activated receptor gamma. Mol Cell Biol 27: 803–17. 17101779 10.1128/MCB.00601-06 PMC1800691

[46] Lombardi A, Cantini G, Mello T, Francalanci M, Gelmini S, et al (2009) Molecular mechanisms underlying the pro-inflammatory synergistic effect of tumor necrosis factor alpha and interferon gamma in human microvascular endothelium. Eur J Cell Biol 88: 731–42. 19782427 10.1016/j.ejcb.2009.07.004

[47] von Knethen A, Soller M, Tzieply N, Weigert A, Johann AM, et al (2007) PPARgamma1 attenuates cytosol to membrane translocation of PKCalpha to desensitize monocytes/macrophages. J Cell Biol 176: 681–94. 17325208 10.1083/jcb.200605038 PMC2064025

[48] Zhang WY, Schwartz EA, Permana PA, Reaven PD (2008) Pioglitazone inhibits the expression of inflammatory cytokines from both monocytes and lymphocytes in patients with impaired glucose tolerance. Arterioscler Thromb Vasc Biol 28: 2312–8. 18818415 10.1161/ATVBAHA.108.175687

[49] Wu ZH, Zhao SP, Chu LX, Ye HJ (2010) Pioglitazone reduces tumor necrosis factor-alpha serum concentration and mRNA expression of adipose tissue in hypercholesterolemic rabbits. Int J Cardiol 138: 151–6. 18809217 10.1016/j.ijcard.2008.08.009

